# Organizing Variables Affecting fMRI Estimates of Language Dominance in Patients with Brain Tumors

**DOI:** 10.3390/brainsci11060694

**Published:** 2021-05-25

**Authors:** Monika M. Połczyńska

**Affiliations:** Department of Psychiatry and Biobehavioral Sciences, David Geffen School of Medicine at UCLA, University of California, Los Angeles, CA 90025, USA; MPolczynska@mednet.ucla.edu; Tel.: +1-310-825-6311

**Keywords:** brain tumor, glioma, language dominance, laterality index, language, fMRI, confounds

## Abstract

Numerous variables can affect the assessment of language dominance using presurgical functional magnetic resonance (fMRI) in patients with brain tumors. This work organizes the variables into confounding and modulating factors. Confounding factors give the appearance of changed language dominance. Most confounding factors are fMRI-specific and they can substantially disrupt the evaluation of language dominance. Confounding factors can be divided into two categories: tumor-related and fMRI analysis. The tumor-related confounds further subdivide into tumor characteristics (e.g., tumor grade) and tumor-induced conditions (aphasia). The fMRI analysis confounds represent technical aspects of fMRI methods (e.g., a fixed versus an individual threshold). Modulating factors can modify language dominance without confounding it. They are not fMRI-specific, and they can impact language dominance both in healthy individuals and neurosurgical patients. The effect of most modulating factors on fMRI language dominance is smaller than that of confounding factors. Modulating factors include demographics (e.g., age) and linguistic variables (e.g., early bilingualism). Three cases of brain tumors in the left hemisphere are presented to illustrate how modulating confounding and modulating factors can impact fMRI estimates of language dominance. Distinguishing between confounding and modulating factors can help interpret the results of presurgical language mapping with fMRI.

## 1. Introduction

While functional magnetic resonance (fMRI) has become a useful and widely used method to assess language dominance before brain surgery, the technique has its limitations that need to be considered when interpreting its results. There are numerous variables that can distort the estimates of fMRI language dominance in patients with brain tumors. The variables include, for example, tumor location, tumor grade, tumor volume, prior neurosurgery, seizure history, aphasia presence and severity, patient demographics (handedness), as well as technical aspects of fMRI analysis (e.g., threshold type) [1,2,3,4,5,6,7,8]. While there have been numerous studies demonstrated that these factors can individually disrupt fMRI assessment of language dominance, these variables have rarely been analyzed collectively in patients with brain tumors [9,10,11,12,13]. Further, some variables have rarely been mentioned in the context of neurosurgical patients (e.g., linguistic factors, such as early bilingualism) [14]. As such, there is a need for theoretical work that organizes variables which can affect language dominance in patients with brain tumors, as assessed by presurgical fMRI.

Functional MRI is a non-invasive technique that allows us to observe the working brain. The method is based on the physics of nuclear magnetic resonance and the effects that blood oxygenation has on the magnetic resonance signal. The fMRI signal captures changes in blood flow and blood oxygenation as subjects perform a given task (e.g., a language task). Specifically, stimuli can elicit increased blood flow that is translated to change in the blood-oxygenation-level dependent (BOLD) signal [15,16].

Functional MRI has been clinically validated by numerous studies using direct comparisons with intraoperative language mapping [17] and Wada testing [18,19]. For example, a study by Ishikawa et al. [18] demonstrated that the results between fMRI and Wada in 74 individuals with gliomas were consistent in the vast majority of cases (91.4%). Of the 8.6% inconsistent cases, the two methods suggested dominance in opposite hemispheres in 5.2% of the patients. In 2.7% of the 8.6% cases, fMRI incorrectly identified the right hemisphere as dominant for language in right-handed patients with gliomas in the left hemisphere. The results were further validated with awake surgery in 34 patients. The authors concluded that language dominance could be successfully determined with fMRI. The exception is patients in whom fMRI suggests atypical language organization or results are inconclusive [18,19].

Hemispheric dominance in fMRI is typically evaluated using the Laterality Index (LI) [20]. In each hemisphere, we calculate the number of voxels active during a language task. A classic formula to compute the LI is (L − R)/(L + R), where L stands for the left hemisphere and R represents the right hemisphere [21]. Results of the LI span between +1 (strong left dominance) and −1 (strong right dominance). As will be presented below, different approaches to an fMRI analysis can significantly alter the LI values in a single subject. The approaches include using threshold-dependent versus threshold-independent calculations [22,23], applying a full-hemisphere versus an region of interest (ROI) approach [24,25], and administering a single task versus a panel of language tasks [11].

The goal of this work is to organize the variables that affect language dominance in patients with brain tumors, as assessed by presurgical fMRI.

## 2. Organization of Variables Affecting fMRI Estimates of Language Dominance in Patients with Brain Tumors

### 2.1. Confounding vs. Modulating Factors

This work organizes multiple variables that can affect the assessment of fMRI language dominance in patients with brain tumors into two broad categories: *confounding factors* and *modulating* *factors*. Confounding factors give the appearance of changed language dominance, whereas modulating factors can actually cause a change in language dominance.

Table 1 illustrates the main differences between confounding and modulating factors. Confounding factors can increase the risk of drawing incorrect conclusions about fMRI language dominance in patients with brain tumors. For the most part, confounding factors are fMRI-specific and they would not distort other methods examining language dominance (e.g., the Wada test). Their impact on the evaluation of fMRI language dominance can be substantial. As presented in Table 2, confounding factors divide into tumor-related and fMRI analysis confounds. The tumor-related confounds include tumor characteristics (e.g., tumor location, tumor grade, tumor volume and the age of tumor onset) and tumor-related conditions (the presence of aphasia, prior neurosurgery). The fMRI analysis confounds represent technical aspects of fMRI methods (e.g., a fixed versus an individual threshold) that can distort the assessment of language dominance.

Modulating factors can modify (but not distort) language dominance in general. As shown in Table 1, factors are not fMRI-specific and they can affect language dominance both in healthy individuals and neurosurgical patients. The impact of most modulating factors on fMRI language dominance is suggested to be smaller than that of confounding factors. Modulating factors include demographics (e.g., age), and linguistic factors (e.g., early bilingualism, sign language) (see Table 2).

### 2.2. Confounds Affecting fMRI Language Dominance

#### 2.2.1. Tumor-Related Confounds

The presence of a brain tumor around the language network can disrupt the assessment of language dominance with fMRI [13,26,27]. Based on prior research [1,12,28,29], it is suggested that fMRI estimates of language dominance can be distorted (usually decreased) by two subcategories of tumor-related confounds: (a) tumor characteristics [1,12,28,29], and (b) tumor-induced conditions [30,31,32]. These two subcategories are presented in Table 2.

##### Tumor Characteristics

Five major tumor characteristics have been reported to disrupt fMRI language dominance:

(a) *Hemisphere (lesion side)*—right-handed patients with tumors in the left language-dominant hemisphere have been observed to have decreased fMRI language dominance [13,26]. On the other hand, tumors in the right hemisphere appear to have a minimal impact on the cortical representation of language. In fact, individuals with right hemisphere tumors have been reported to have similar language activations to those observed in healthy controls [12]. The decreased dominance for language in patients with tumors in the left-dominant hemisphere has been associated with reduced activity in language regions proximal to the lesion [2,3]. The diminished fMRI activation has also been attributed to tumor neovasculature (neurovascular uncoupling) [1,26,33,34]. Concurrently, right homologs of tumor-affected areas in the left hemisphere may show increased BOLD signal during language tasks due to functional compensation or pseudo-reorganization [3,27,28,32].
(b) *Tumor location in the grey matter of the language-dominant hemisphere*—brain tumors around the anterior language regions have been associated with less robust fMRI language dominance than tumors around the posterior language areas [1,12]. Other fMRI studies did not confirm these findings [11,24]. However, the results of the studies are difficult to compare due to differences in the methodological design, including, for instance, not matching samples based on confounding factors that may have biased the results. A recent study [25] addressed these challenges. Sixty patients were matched on a case-by-case basis to control for 11 confounding and modulating factors known to affect fMRI estimates of language dominance (e.g., tumor grade, patient demographics). Consistent with the results by Gohel et al. [1] and Wang et al. [12], the recent study found that brain tumors around Broca’s area decreased fMRI language dominance independent of known confounds. Conversely, tumors affecting Wernicke’s area did not significantly distort fMRI language dominance. The fMRI values of language dominance in these patients were similar to those observed in patients with tumors in the right hemisphere [25].
(c) *Tumor grade*—patients with low-grade tumors are more likely to show functional reorganization because their tumor progression is typically more extended. In such cases, plastic changes helping preserve language function are thus more likely to occur [3,27]. For example, a low-grade tumor can cause gradual destruction of Broca’s region with a concurrent reorganization (“rewiring”) to other areas. The reorganization is usually directed away from the tumor to compensate for a functional language loss [12,32,35,36]. Specifically, function redistribution can occur in areas surrounding the tumor [37], more distant regions in the same hemisphere [38], or contralesionally, in areas homologous to the lesioned structure [39,40]. Lower fMRI language laterality before brain surgery has been positively correlated with a smaller risk of transient and permanent aphasia in some studies [4], but not others [28]. Pseudo-organization, on the other hand, is frequently reported in patients with fast-growing, high-grade gliomas in whom the tumor disinhibits the contra-lesional hemisphere. Because eloquent language regions affected by the tumor do not have sufficient time to move away from the glioma in these patients, the increased right hemisphere activity is not associated with good functional performance [33]. For instance, Fernández-Coello et al. [5] described a patient suffering from a small but aggressive glioblastoma. The authors noted an area with language activity within the extension of the tumor. They concluded that the active region did not have enough time to relocate further away from the tumor [5].

There is an additional aspect that may differentiate an fMRI assessment of language dominance in patients with low versus high-grade brain tumors. Specifically, patients with high-grade tumors may have abnormal vasoreactivity with resultant neurovascular uncoupling in regions proximal to tumors. In consequence, these patients may not respond as robustly to the increase in neural activity, which may result in a muted BOLD response or false negatives [41,42]. While neurovascular uncoupling is more frequent in individuals with high-grade tumors, it can also occur in patients with low-grade tumors [43].

(d) *The age of tumor onset*—this characteristic is often colinear with tumor grade: low-grade, slow-growing tumors typically appear during childhood, while fast-growing, aggressive tumors (e.g., glioblastomas) are more likely to occur in adulthood [6]. It is known from epilepsy studies that language may be atypically organized in individuals with a pediatric epilepsy onset [44]. According to Lidzba et al. [29], the window of time for language to reorganize successfully after insult to the left language-dominant hemisphere is before the age of five. Cases with brain lesions within this time span always had a favorable outcome in terms of language function. These patients had either bilateral or right-dominant language organization [29]. Conversely, lesions that occurred after the age of five had an unfavorable outcome in the majority of patients. Those patients presented moderate to severe impairments in language. None of the patients displayed the reorganization of language function to the contra-lesional right hemisphere [29]. It is still debatable whether language reorganization can take place in individuals with brain damage later in life. It seems that functional compensation is more likely in such cases. For example, Teki et al. [45] showed that adult stroke patients with disrupted language comprehension relied more on the right hemisphere (specifically, the right auditory regions, including the superior temporal gyrus) when processing speech. While the shift to the right hemisphere might have been compensatory in nature, the patients remained considerably impaired. While functional compensation of language later in life is possible, its magnitude is generally significantly smaller when compared with functional reorganization seen in pediatric patients [29,45,46].
(e) *Tumor volume*—this characteristic has been researched less extensively than the tumor characteristics discussed above. Tumor size might impact language laterality if it is located in the language-dominant hemisphere. There is scarce information on the impact of volume of high-grade tumors on fMRI language dominance. Yet, it is plausible that this variable may disrupt fMRI language laterality in high-grade tumors to a much greater extent than in low-grade tumors. One study demonstrated that a larger size of high-grade gliomas was associated with poorer performance on executive functioning tests [47]. Fernández-Coello et al. [5] presented several cases that illustrate a less pronounced impact of tumor size in patients with low-grade tumors. One of their patients had a large, (seven cm in diameter) slow-growing brain tumor in the dominant left hemisphere. As indicated with preoperative fMRI, the patient’s language was organized bilaterally. Yet, no functional language regions were identified during intraoperative language mapping. The authors suggested that functional reorganization had taken place in this case. No eloquent language sites were found in areas where language was expected otherwise. Thus, the sizable tumor had likely triggered a shift in language laterality without causing language disruption. Fernández-Coello et al. [5] described two additional cases of slow-growing large tumors that also displayed functional reorganization.

Understanding the impact of tumor location in the grey versus white matter can be another helpful characteristic in interpreting language activity and hemispheric dominance as depicted by fMRI. Whereas grey matter has a considerable plastic potential, plasticity in subcortical regions is low. The limited plasticity of the white matter pathways has been demonstrated by stroke studies in which damage to fiber bundles generated more profound neurological deficits than damage to the cortex [48]. There are strong implications that brain plasticity is possible only with preserved subcortical connectivity [48,49,50]. Thus, in patients with brain tumors affecting subcortical regions subserving language (e.g., the arcuate fasciculus or the inferior fronto-occipital fasciculus), one can expect less functional compensation and a higher potential to develop language impairments. In such cases, lower language dominance values on fMRI language tasks due to elevated activity in the right hemisphere could indicate pseudo-reorganization, particularly with a concurrent aphasia diagnosis [51]. Yet, further research is required to determine the difference between tumors located in the grey versus white matter, as well as tumors that span through both of these structures.

Another possible tumor characteristic that may disrupt fMRI language dominance is whether a brain tumor is infiltrating or fully encapsulated. Infiltrating tumors might distort fMRI language dominance to a higher degree than fully encapsulated tumors. However, more research is needed to advance our understanding of this tumor characteristic on fMRI language dominance.

##### Tumor-Induced Conditions

Tumor-induced conditions can occur subsequent to the presence of a brain tumor, and they include:

(a) *Aphasia*—disrupted language comprehension and/or production has been reported in between 10.4 to 36.4% of individuals with brain tumors residing in the language-dominant left hemisphere [30,52,53,54,55]. The vast discrepancies in aphasia reports may result from methodological differences in patient selection and assessments between studies, such as including patients with both low and high tumor grades or enrolling patients with recurring tumors [30]. In general, patients with low-grade gliomas have milder or no language impairments compared to high-grade gliomas. A faster rate of growth of high-grade brain tumors may not provide sufficient time to recruit other brain structures to compensate for language disruption [30,56,57]. Patients with brain tumors who suffer from aphasia have been found to have reduced BOLD signal during resting-state fMRI in the left inferior frontal gyrus when compared with tumor patients with no aphasia who displayed retained BOLD signal in this area [56]. Therefore, language dominance, as evaluated with fMRI, might be less robust in individuals diagnosed with aphasia. While language disruption usually occurs when a brain tumor is located within the language network, tumors outside the classical language regions may also cause language disruption, as shown by several studies using intraoperative language mapping [55,58,59].
(b) *Previous neurosurgery*—prior brain surgery has been demonstrated to disrupt the BOLD signal by image distortions and signal dropout in regions that are in the vicinity of the surgical zone [31,60]. By obscuring actual language activation, previous neurosurgery is assumed to be a significant confounding factor in clinical language mapping [31]. A recent study [25] compared patients with a history of prior surgery who were diagnosed with recurrent tumors and patients with tumors without prior surgery. The patients all had their tumors around the left inferior frontal gyrus of the language-dominant left hemisphere (Brodmann areas 44/45/47), as determined clinically. The patients with prior surgery had lower values of fMRI language dominance with more activity in the right hemisphere, as compared with the patients with no prior surgery. The amount of activity in the left hemisphere was not significantly different between the two samples. The authors replicated these findings with a region of interest (ROI) approach for the affected Broca’s region but not the unaffected Wernicke’s region. In the latter area, laterality values were equal in both groups. Thus, previous surgery needs to be considered in patients who underwent prior resections and need to undergo preoperative language fMRI because they anticipate another surgery [25,51]. In these individuals, using an ROI approach within unaffected areas of the language network is recommended [25]. Such patients constitute a significant portion of all neurosurgical patients with brain tumors. In one medical center, as many as a quarter of all patients referred for presurgical mapping had previous neurosurgery [31].

An additional confounding factor in the category of tumor-induced conditions could be the history of seizures. Seizures are a common phenomenon in patients with gliomas. They are the most likely to develop in individuals with low-grade gliomas. In contrast, fewer than 50% of individuals with high-grade gliomas, as well as brain metastases, experience seizures [61]. Although there has not been much data on the effect of seizures on fMRI language dominance, it is assumed that seizures might also constitute a confounding factor. One longitudinal study using navigated repetitive transcranial magnetic stimulation demonstrated that patients with gliomas in the left hemisphere suffering from seizures were more likely to display a decrease in language dominance when compared with glioma patients with no seizures [32]. Yet, more research is required to validate the effect of seizures on language dominance.

Another tumor-induced confounding factor that could disrupt fMRI language dominance is edema of the brain [25]. Nevertheless, more research is needed to understand how edema (especially dense and large) can affect fMRI signal during language tasks.

#### 2.2.2. The Confounds of fMRI Analysis

The confounds of fMRI analysis represent several technical aspects of fMRI methods which can alter the values of language dominance (see Table 2). The factors include:

(a) *Threshold*—the values of fMRI language dominance can change with the statistical threshold [7,20]. Using a fixed threshold could render voxel activation differences between the hemispheres [23]. Consequently, language dominance may be calculated suboptimally [9,62]. Ruff et al. [7] reported that the values of fMRI language dominance in individuals with brain tumors were considerably more sensitive to changes in threshold values than in healthy controls. Using a fixed threshold in clinical practice can make the right hemisphere seem as dominant as the left hemisphere, but such an interpretation may be incorrect [11]. Not surprisingly, a survey showed that 79% of 63 epilepsy surgical programs used statistical thresholding that varied by the patient [63]. Several approaches have been proposed to calculate threshold-independent LI in the clinical setting, including a recent simplified method of LI calculation [22].
(b) *Number and type of language tasks*—lateralization patterns of language may change depending on the type of task used [11,13,64]. For example, a word generation task can produce higher laterality values than a reverse reading task [13]. Several studies have demonstrated that applying multiple language tasks in presurgical fMRI improves the evaluation of hemispheric dominance by increasing its power [65,66]. A multitask approach is considered to be superior to a single task approach because the former accounts more precisely for the complexity of language function [14,65,67,68].
(c) *Region of interest versus whole-brain approach*—several studies reported that using an ROI approach showed more robust fMRI estimates of language dominance than a whole-brain approach in patients with brain tumors [25] and in healthy controls [23,69]. It is important to examine within-hemisphere variations that can occur in fMRI activation preoperatively. The variations can help determine not only how each hemisphere, but also how each area within the language-dominant hemisphere contributes to the language process [69,70]. Using an ROI approach can be particularly helpful in individuals with brain tumors affecting Broca’s area because these patients have been demonstrated to display less robust fMRI language dominance in this region. In these patients, applying an ROI approach using posterior language regions was recommended because fMRI language laterality was not distorted in those areas [25].

A few other factors have been suggested to affect fMRI language dominance, including, for instance, the calculation strategy of language laterality, and fMRI analysis of z-values [13,71]. These variables should also be considered as potential confounding factors that might affect fMRI language dominance.

### 2.3. Factors Modulating Language Dominance

#### 2.3.1. Patient Demographics

The following characteristics can modify fMRI language dominance in the general population and in patients with brain tumors (see Table 2):

(a) *Handedness*—the majority of individuals who are left-hemisphere dominant for language also present greater right-hand dexterity [72,73,74]. Concurrently, right-handed individuals display considerable interindividual differences, and most show some degree of activation in the right hemisphere [44,75]. Left-handers are more likely to have atypical language lateralization (around 30%), compared to right-handers (around 5% to 7.5%) [76,77,78]. Atypical right-hemisphere dominance does not seem to mirror the language organization of the typical left-hemisphere dominance [79]. Left-handers have been demonstrated to be more heterogeneous in language lateralization and performance compared to right-handers [80,81,82]. Finally, resting-state fMRI has revealed that left-handed individuals have a higher degree of interhemispheric intrinsic connectivity between the left and right language networks, coupled with a larger volume of the corpus callosum [79]. Thus, left-handedness can be a robust factor that can modulate language dominance in both healthy controls and neurosurgical patients. At the same time, one study [8] that examined patients low-grade gliomas found that, while the right hemisphere may participate in language processing, the left hemisphere still played an essential role in language. In that study, ten left-handed individuals underwent intraoperative electrical language mapping in the left hemisphere. In nine patients, intraoperative language distortions were induced on a cortical and subcortical level (e.g., the lateral segment of the superior longitudinal fasciculus, arcuate fasciculus) [8].
(b) *Age*—although the impact of age on language dominance is not completely understood, generally, left-lateralization increases with age in children and begins to gradually decline in adulthood [76,83,84]. More specifically, Szaflarski et al. [84] used a verb generation task in fMRI to investigate 170 right-handed children and adults. The authors found that language lateralization increases between five and 20 years of age, with the left hemisphere being dominant in most cases. Then, language laterality plateaus between the ages of 20 to 25. Finally, it slowly starts to decrease between age 26 and 70 [84]. The latter observation has been confirmed in adult individuals with brain tumors in whom fMRI dominance values for language tended to decrease with age [13]. One model accounting for the gradual drop in language laterality values as we age is the hemispheric asymmetry reduction in older adults (HAROLD) model [85]. The model assumes less laterality when older individuals perform cognitive tasks in comparison to younger adults [85].
(c) *Gender*—it appears that differences in language laterality between males and females exist, but they may be dependent on age [84,86,87]. For instance, Nenert et al. [87] observed that language laterality values drop with age significantly, but only in the temporo-parietal area and only in right-handed men. The authors postulated that there might be different developmental trajectories of language laterality in men and women, as well as right- and left-handers (the authors found no significant changes in language laterality both in older women and in right-handed men) [87]. Another study [88] examined 22 women and 25 men with brain tumors. During language tasks, the females displayed fMRI activations within the areas associated with language. In contrast, activations in men extended the classical language network (e.g., the precentral gyrus and supplementary motor area).

It is important to highlight the fact that—while the effect of left-handedness on fMRI language dominance can be robust [76,77,79]—the impact of age and gender appears to be much more subtle. In some cases, the two variables can also be interdependent. Moreover, even when we account for handedness, age, and gender, there is still a considerable amount of individual variability in fMRI language mapping that has been shown in healthy volunteers [89]. The variability may be linked to individual factors, including, for instance, language performance and verbal IQ [90]. We do not quite understand the relationship between the estimate of language dominance as shown by fMRI and language performance in healthy individuals because only a few studies with mixed findings have investigated this association. As for verbal IQ, one study [90] found that there was a positive correlation between language dominance and verbal IQ during an fMRI task on vowel detection.

#### 2.3.2. Linguistic Factors

The properties of languages themselves should also be considered when interpreting fMRI language dominance results. The three modulating factors described below have been linked to decreased values of language dominance in healthy individuals and individuals with brain tumors, using fMRI:

(a) *Early bilingualism*—a few studies on healthy volunteers have indicated that early bilinguals have a more bilateral representation of their languages compared to late bilinguals and monolinguals [91]. Decreased fMRI language dominance was also reported in a study involving 25 early bilinguals with brain lesions (including brain tumors), and 25 matched monolingual patients undergoing presurgical fMRI [14]. Language dominance was decreased in both the first (L1) and the second (L2) language. Importantly, it was a more pronounced engagement of the right hemisphere in each of the languages that resulted in less pronounced left-language dominance in the early bilingual patients. The volume of language activation in the left-dominant hemisphere in L1 was the same in those patients as in the monolingual controls [14]. Moreover, a study [92] involving bilingual patients with brain tumors found that fMRI language mapping performed in both languages provided a superior appreciation of the language network than mapping in only one language (i.e., more language-related regions were identified). This finding has been confirmed by a recent systematic review of studies with patients (including individuals with brain tumors) who underwent neurosurgical language mapping (pre-operative fMRI, Wada, and/or electrical stimulation mapping) [93].
(b) *Language characteristics*—in comparison to most languages from the Indo-European family (e.g., English, French, Spanish), some non-Indo-European languages have been associated with weaker fMRI language dominance [94]. In particular, more involvement of the right hemisphere has been observed in several Asian languages. For example, elevated right hemisphere activation has been found during reading logosyllabic characters [95] and processing lexical tones in Chinese [94,96].
(c) *Sign language*—language modality can be signed or spoken. Both modalities recruit the language network in the left-dominant hemisphere [97,98,99]. At the same time, sign and spoken language use different input modalities in the language system: sign language uses a visuospatial modality, whereas spoken language employs an auditory modality. The main differences in language laterality between sign and spoken language have been ascribed to their input modalities [99]. Those differences seem prominent in language comprehension, where sign language has been associated with additional recruitment of the right hemisphere compared to spoken language [100,101]. The production of sign language has been demonstrated to be strongly left-lateralized, and it lacks the additional recruitment of the right hemisphere observed during sign language comprehension [99,102]. Therefore, in the context of neurosurgery, language dominance can be expected to be less left-lateralized in tasks assessing the comprehension of sign language.

## 3. Cases

This section presents three patients with tumors in the left hemisphere located within the language network. These retrospective cases illustrate how various modulating and confounding factors and confounds can impact fMRI estimates of language dominance. Data presented in this section come from research that was approved by the University of California, Los Angeles (UCLA) Institutional Review Board.

All the patients underwent presurgical language fMRI using a 3T Prisma or Allegra scanner (20- and 12-channel head coils, respectively; Siemens, Malvern, PA, USA). A T2 image was acquired for each patient using these parameters: voxel size = 0.5 × 0.5 × 3 mm^3^, 90° flip angle, TE = 6670 ms, TE = 58 ms, field of view = 200 mm, matrix dimensions 263 × 350 mm^2^, turbo spin echo, and generalized autocalibrating partial parallel acquisition (acceleration factor = 2). Next, echo-planar image parameters were applied, including voxel size = 3.1 × 3.1 × 3 mm^3^, 90° flip angle, TE = 53 ms, TR = 2500 ms, 28 slices, 90 volumes, matrix dimensions = 200 × 200 mm^2^, and field of view = 200 mm. 

The patients performed three fMRI language tasks: object naming, auditory responsive naming, and verbal responsive naming. In the object naming task, the patients were instructed to silently provide a name for a black-and-white object presented on a screen (e.g., a unicorn). In the auditory responsive naming task, the subjects were directed to listen to a phrase and think of the object that was being described to them (e.g., “a barking animal”). In the verbal responsive (reading) task, the patients were asked to read a phrase silently and think of the object that the phrase described (e.g., “people write with it”).

An experienced neuropsychologist created language maps using minimal preprocessing. Task-related activations were identified through the application of a Pearson’s Correlation Coefficient (for details, see [103]). At UCLA, we use custom software for clinical fMRI analysis developed at our center. The software converts raw MRI mages from the DICOM format to paradigm (.bfloat) and image (.bshort) files. The data is examined visually for potential artifacts, including, for example, excessive noise, ring artifacts, steal artifacts, and radio frequency interference. Since patients are typically administered each language task twice, we can select a task run that has superior quality. We rarely use motion correction. With interleaved acquisition that was applied, if there is a within-TR motion, the motion correction algorithms (which work at the whole volume level) do not account for within-TR motion. Clinicians at UCLA do not analyze data with significant head motion because of a risk of spurious activations or negative findings. In case of a clear pattern of motion in a task, such as a shift in the head position toward the end of a task, blocks affected with motion are excluded from the analysis. 

Language-related activations are determined with Pearson’s Correlation Coefficient (for details, see Benjamin et al. [103]). Briefly, data smoothing was conducted using a 2 mm Gaussian kernel. By applying a minimal smoothing kernel, noise was reduced but accurate anatomical localization was not obscured. A regressor that included the expected time series was convolved with a hemodynamic response function. A correlation between expected and actual activation was taken. Data were inspected for quality. If two runs of a language task were completed, the run judged superior was applied in the further analysis. Every language map was first thresholded with a correlation of r = 0.2. The threshold was then adjusted individually for each patient. The adjustment was performed until an optimal organization of functional language sites was identified (applying an individual threshold for every patient has been clinically validated as superior to using a fixed threshold [23]). Using an effective significance value (*p* < 0.000123 (0.05^3^)), a conjunction of the language maps was performed. Based on the Bayes theorem [104], this approach is non-task specific, and it minimizes non-language activation, such as sensory activation (e.g., activation in the visual cortex during an object naming task) [25]. In comparison to other evaluations of language dominance, the approach has been demonstrated to be valid, systematic, and reliable [103].

### 3.1. Case 1

**Figure 1:**
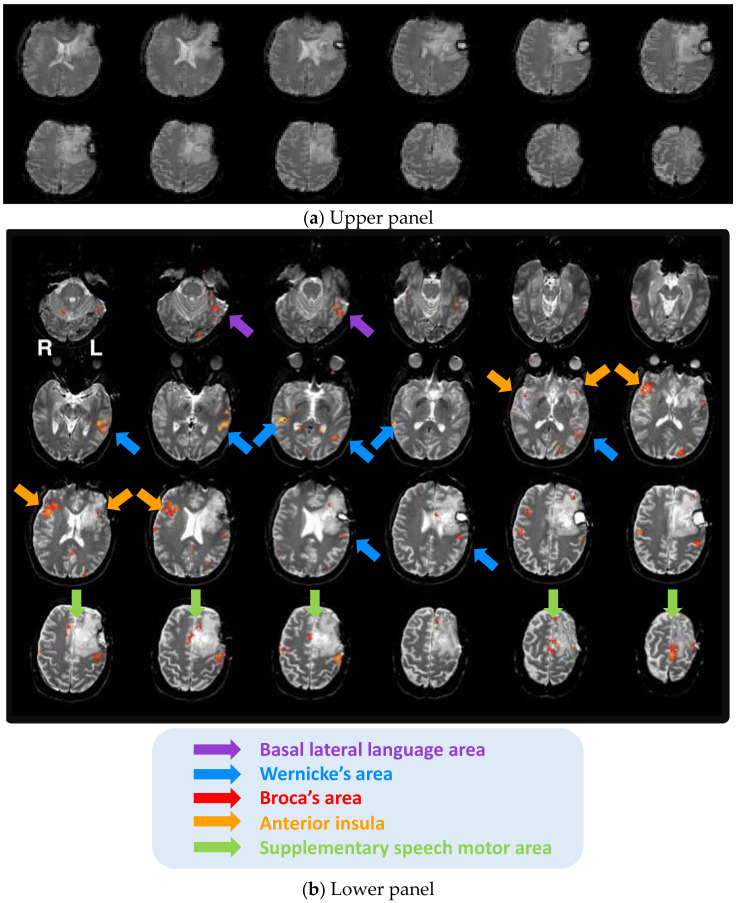
Disrupted fMRI language dominance in Case 1. (**a**) Upper panel: signal loss in and around the lesion from prior resection. (**b**) Lower panel: the lack of functional activity associated with Broca’s area can be observed.

Case 1 is a 37-year-old, right-handed male with left frontal glioblastoma (World Health Organization; WHO grade IV). The patient had a history of prior resection and was under consideration for additional surgery to address tumor regrowth when he received his language fMRI exam. The patient was diagnosed with moderate expressive aphasia. There was a significant loss of signal in and around the lesion from prior resection. This signal loss was located approximately where Broca’s area should be represented. The upper panel of Figure 1 shows the raw fMRI. The area of prior resection was filled with the cerebral spinal fluid (bright white color). There was clear signal loss due to which the functional cortex associated with Broca’s area could not be observed (the lower panel of Figure 1). 

An infiltrating nature of the tumor, as well as the patient’s expressive language deficits may have further disrupted language activation in this region. The patient displayed increased activation of the motor cortex, including the insular motor area in the right hemisphere, bilateral activation of the tongue motor area, and medial pre-motor activation to the right of midline consistent with the supplementary speech motor area and speech motor area proper activation. These elevated motor activations might be interpreted as compensatory mechanisms following the language deficits (see Figure 1 for details). While no language activity was noticed in Broca’s region, eloquent language sites were reported in this area during subsequent intraoperative language mapping both cortically and sub-cortically (because of the infiltrative nature of the patient’s tumor, resection was carried out, while the patient engaged in ongoing language testing). In sum, numerous confounding factors may have contributed to disrupted fMRI language dominance in Case 1: an anterior tumor location affecting both the grey and white matter, high tumor grade, likely adult (more recent) onset, large tumor volume, infiltrating nature of the tumor, aphasia, prior surgery, and seizures. Table 3 summarizes the confounding and modulating factors for Case 1.

### 3.2. Case 2

**Figure 2:**
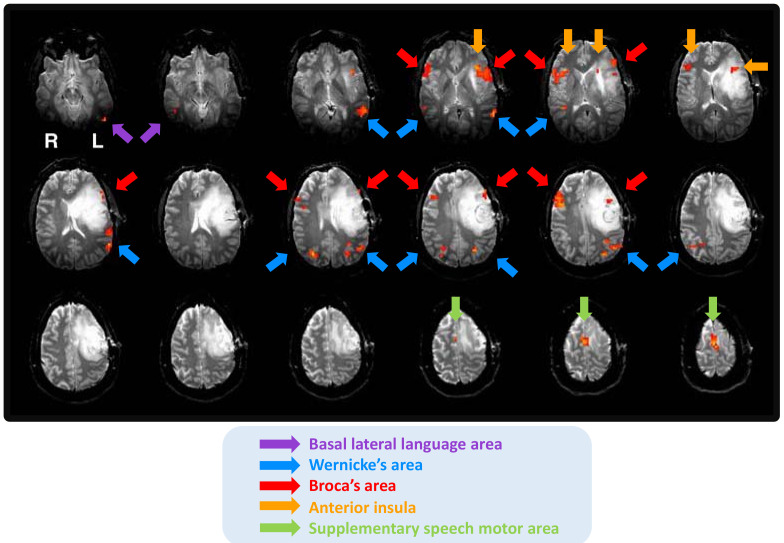
Bilateral language activations in Case 2.

Case 2 is a 38-year-old, right-handed female diagnosed with WHO grade III frontal glioblastoma in the left hemisphere. Despite a large volume of her lesion, the patient did not suffer from any language impairments. Language appeared to be left-hemisphere dominant in this patient but with a bilateral representation of Broca’s area and the superior part of Wernicke’s area (see Figure 2). 

The patient underwent intraoperative language mapping. Eloquent language regions were identified during the procedure. No data on postoperative outcome was available. Several confounding factors may have disrupted fMRI language dominance in Case 2: anterior tumor location affecting both the grey and white matter, high tumor grade, likely adult-onset, a large tumor volume, infiltrating nature of the tumor, and the history of seizures. It is possible that language was bilaterally organized in this patient premorbidly. This assumption is made based on considerable activations in the right anterior regions. Even more importantly, Case 2 suffered from no language impairments in spite of her tumor characteristics that are typically associated with a higher risk of language deficits. Confounding and modulating factors for Case 2 are presented in Table 3.

### 3.3. Case 3

**Figure 3:**
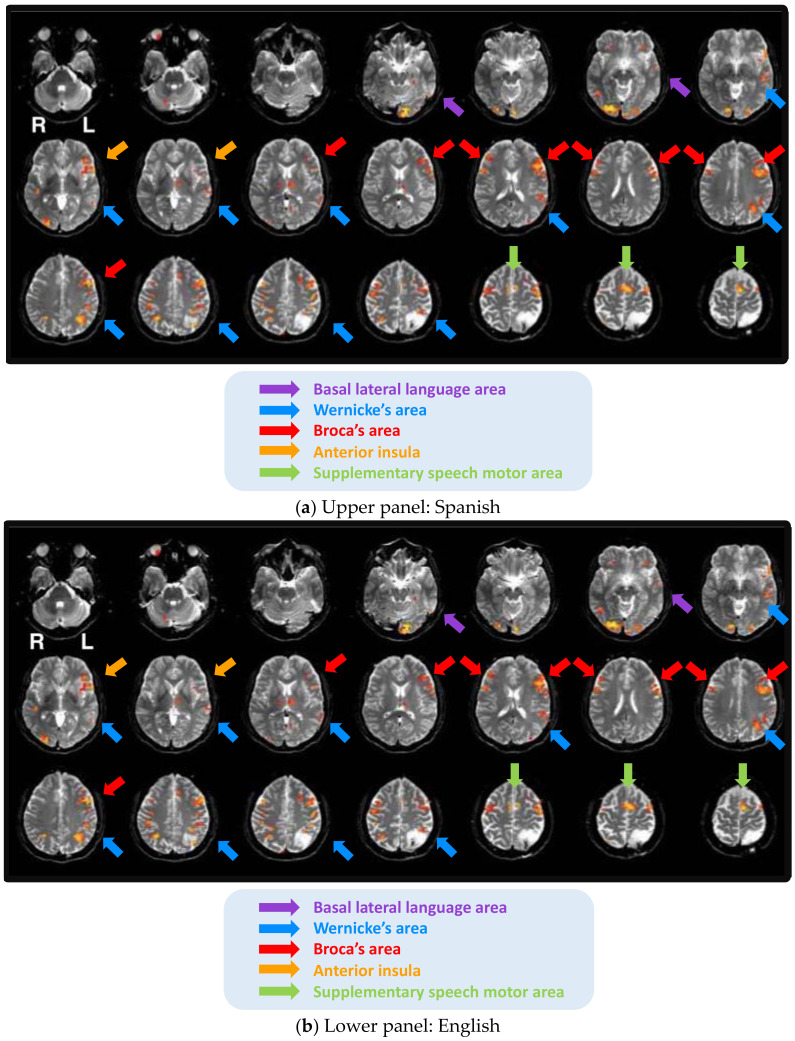
Language activity in Spanish and English in Case 3.

Case 3 is a 58-year-old left-handed female. The patient’s biopsy was consistent with WHO grade II anaplastic astrocytoma in the left parietal lobe. The patient was an early bilingual with Spanish as her first and primary language and English as her second language. She performed fMRI tasks in both of her languages. As presented in Figure 3, Spanish and English were left hemisphere dominant, with some bilateral representation of Broca’s area in both languages and the basal temporal language area in Spanish.

The patient underwent awake surgery, during which the functional cortex was identified. No postoperative language aphasia was diagnosed. The patient’s language abilities were not compromised, possibly because she had a low-grade glioma that was relatively small in size and was located superior to the key language areas. Her increased right hemisphere activity may have been due to the fact that the patient was a left-handed, early bilingual. In addition, she suffered from a low-grade glioma, which may have enabled functional compensation. All of these factors may have contributed to an elevated right-hemisphere activity [14,76,77]. Moreover, the tumor was located in the grey matter and its resection did not affect white matter structures. Confounding and modulating factors for Case 3 are presented in Table 3.

## 4. Discussion

This work organizes multiple variables that can affect the assessment of fMRI language dominance into two broad categories of confounding and modulating factors. Confounding factors give the appearance of changed language dominance, whereas modulating factors can cause an actual change in language dominance. Most confounding factors are fMRI-specific, and other methods examining language dominance (e.g., the Wada test) would not be distorted by the confounding factors. The impact of confounding factors on the evaluation of fMRI language dominance can be substantial. There are tumor-related and fMRI analysis confounds. The tumor-related confounds include tumor characteristics (e.g., tumor location, tumor grade, volume and the age of onset) and tumor-induced conditions (the presence of aphasia, prior neurosurgery). The fMRI analysis confounds represent technical aspects of fMRI methods (e.g., a fixed versus an individual threshold) that can also disrupt the assessment of language dominance. Modulating factors can modify language dominance without confounding it. Modulating factors are not fMRI-specific and they can impact language dominance both in healthy individuals and neurosurgical patients. The effect of most modulating factors on fMRI language dominance is suggested to be smaller than that of confounding factors. Modulating factors include demographics (e.g., age), and linguistic factors (e.g., early bilingualism, sign language). Organizing the multiple variables into the two distinct categories that can affect fMRI language dominance can help interpret the results of presurgical language mapping with fMRI, as illustrated with the three cases.

Table 2 summarizes how confounding and modulating factors can affect fMRI estimates of language dominance in individuals with brain tumors. In the category of confounding factors, the following tumor characteristics can decrease fMRI language dominance (see the weaker dominance column in Table 2): a brain tumor in the language-dominant left hemisphere (versus right), an anterior (versus posterior) tumor location within the language-dominant left hemisphere, a fast-growing, high-grade tumor (versus a low-grade tumor), an adult onset (versus a pediatric onset) tumor, and a large (versus small) tumor size (particularly, in high-grade tumors). In the tumor-induced subcategory, the presence of aphasia (versus the absence of aphasia) and previous neurosurgery (versus no neurosurgery) are confounding factors that can decrease the values of fMRI language dominance. Furthermore, a few aspects of fMRI methods can generate lower fMRI language dominance values, including using a fixed threshold (versus an individual threshold), applying a single language task (versus a panel of tasks), and using an ROI approach (versus a whole-brain approach).

Within the category of modulating factors in Table 2, in the subcategory of patient demographics, language dominance can be less robust in left-handed (versus right-handed) individuals, as well as in females (versus males), and in children and older adults (versus younger adults) (note again that both age and gender can have a rather subtle impact on language dominance). Within the subcategory of linguistic factors, three variables can contribute to lower language dominance: early bilingualism (versus late bilingualism and monolingualism), sign language (versus oral language; particularly sign language comprehension), and language characteristics (e.g., certain non-Indo-European languages using lexical tones and non-alphabetic, logosyllabic character reading).

Variables associated with more robust language fMRI dominance are presented in the stronger dominance column in Table 2. Tumor characteristics (e.g., a brain tumor located within posterior language sites, a low-grade tumor) do not increase left-language dominance but, rather they are less likely to decrease it than the tumor characteristics listed on the left side of the figure (e.g., a brain tumor located within posterior language areas, a high-grade tumor). The remaining confounding and modulating factors, on the other hand, have been linked to more robust (fMRI) language dominance [7,25,31,55,65,75,76,87,91,94,100].

Although this work reviews how numerous variables can impact fMRI language dominance in patients with brain tumors, this knowledge has clear practical applications. The information discussed here can help interpret language fMRI results by alerting neurosurgical teams which confounding and modulating factors should be considered when assessing language dominance to evaluate a postoperative risk of language impairment and surgical planning. As illustrated with the three cases of tumors in the left hemisphere, different combinations of confounding and modulating factors can decrease language laterality values assessed with fMRI.

Several variables have been mentioned in this work but have not been included in Table 2, such as tumor location in the white versus grey matter or the history of seizures in patients with brain tumors. It is hoped that future research will provide more data on the impact of these variables on fMRI language dominance to incorporate them into the proposed two categories of confounding and modulating factors. There are also additional questions, the answers to which could help us further understand how brain tumors impact language dominance. For instance, we still do not know what the critical amount of distortion (deviation from the normal brain) is required for variations in language dominance to start showing. We also do not understand well how close the lesion needs to be to a language site for functional compensation to take place.

Finally, while this work is devoted to task-based fMRI used in the context of presurgical language mapping, it is important to mention resting-state fMRI. Resting-state fMRI has the potential to address the limitations of task-based fMRI [105]. The limitation of task-based fMRI include a high degree of compliance with scanning procedures (e.g., the ability to perform lengthy language tasks and staying still in the scanner). These requirements often cannot be met by pediatric patients, or individuals suffering from tumor-induced language deficits, among others. In contrast, the only requirement in resting-state fMRI is for the subject to stay awake, relax, look at a cross-hair (or have eyes closed), and not think about anything in particular [106]. Thus, resting-state fMRI can potentially map the language network in patients with whom task-based fMRI is challenging or not possible at all [107].

Language maps obtained with resting-state fMRI are less lateralized than those obtained with task-based fMRI [108,109]; although a few studies disagree with this finding [110]. A recent work [108] explained that the more symmetric language maps for resting-state fMRI were the result of more bilateral activations in the anterior (inferior frontal gyri) and posterior (temporal gyri) regions. Concurrently, task-based fMRI generated maps that displayed areas which are not specific to language (e.g., bilateral middle frontal gyrus and bilateral rostral cingulate zone) [108]. The increased restriction to the critical language areas in resting-state fMRI as compared with task-based fMRI was confirmed by other studies also [110]. A review of 45 resting-state fMRI studies in patients with gliomas [111] demonstrated that the technique has a potential clinical utility for presurgical language mapping. At the same time, the authors cautioned that resting-state fMRI language maps could be disrupted by diffuse changes in functional connectivity occurring both locally and inter-hemispherically. More specifically, the functional changes involved increased local efficiency and decreased long-distance connectivity. In addition, tumor grade was correlated with alternations in functional connectivity [111]. It is argued that the confounds and modulating factors may impact the assessment of resting-state fMRI language maps in patients with brain tumors [56,79]. However, more research is required to determine the impact of specific confounding and modulating factors on language maps obtained with resting-state fMRI compared task-based fMRI (e.g., studies elucidating the mechanisms through which brain tumors induce changes in brain connectivity patterns [111]).

## 5. Conclusions

This work organizes multiple variables that can impact the assessment of fMRI language dominance into two broad categories of confounding and modulating factors. Confounding factors give the appearance of changed language dominance, whereas modulating factors can cause an actual change in language dominance. Organizing the multiple variables into the two distinct categories that can affect fMRI language dominance may help interpret the results of presurgical language mapping with fMRI in patients with brain tumors. It is recommended that future research should further examine the impact of the variables that were mentioned only briefly in this work because of insufficient results to make more definite conclusions (e.g., the history of seizures). It is hoped that the influence of confounding and modifying factors will be studied more extensively in the context of within-hemisphere localization. Finally, there is a need for more studies to determine the role of the confounding and modifying factors using presurgical resting-state fMRI in patients with brain tumors.

## Figures and Tables

**Table 1:** Differences between confounding and modulating factors in affecting fMRI estimates of language dominance.

Characteristics	Confounding Factors	Modulating Factors
fMRI specific	Yes	No
Disrupts the assessment of fMRI language dominance	Yes	No
Individuals affected:		
Patients with brain tumors	Yes	Yes
Healthy individuals	No	Yes
The magnitude of impact on fMRI language dominance:		
Large	No	Yes
Small	Yes	No

**Table 2:** Confounding and modulating factors affecting fMRI estimates of language dominance in patients with brain tumors. * in the language-dominant hemisphere; ** variables having only a subtle impact on fMRI language dominance.

Variable	Variable Type	Weaker Dominance	Stronger Dominance
**Confounding factors**	Tumor-related		
	Tumor characteristics		
	Hemisphere	Left	Right
	* Location	Anterior	Posterior
	* Grade	Low	High
	* Age of onset	Adult	Pediatric
	* Volume	Large	Small
	Tumor-induced conditions		
	Aphasia	Yes	No
	Previous surgery	Yes	No
	Technical aspects of fMRI analysis		
	Threshold	Fixed	Individual
	Tasks	Single	Panel
	Analysis	Whole-brain	ROI
**Modulating factors**	Patient demographics		
	Handedness	Left	Right
	** Age	Younger adults	Children, older adults
	** Gender	Male	Female
	Linguistic Factors		
	Bilingualism	Early bilinguals	Mono-/late bilinguals
	Language modality	Signed	Oral
	Language characteristics	Tones	No tones
		Non-alphabetic	Alphabetic

**Table 3:** Confounding and modulating factors in three patients with brain tumors in the left hemisphere.

Variable	Variable Type	Variable Specifics	Case 1	Case 2	Case 3
Confounding factors	Tumor characteristics	Hemisphere	Left	Left	Left
		Location (anterior vs posterior)	Anterior	Anterior	Posterior
		Grade	IV	III	II
		Age of onset	Adult/recent	Adult/recent	Likely not recent
		Volume	Large	Large	Small
	Tumor-induced conditions	Aphasia	Yes	No	No
		Previous surgery	Yes	No	No
	Technical aspects of fMRI analysis	Threshold	Individual	Individual	Individual
		Tasks	Panel	Panel	Panel
		Analysis	Whole-brain	Whole-brain	Whole-brain
Modulating factors	Patient demographics	Handedness	Right	Right	Left
		Age	37	38	58
		Gender	Male	Female	Female
	Linguistic factors	Bilingualism	Monolingual	Monolingual	Early bilingual
		Language modality	Oral	Oral	Oral
		Language characteristics	No tones, alphabetic	No tones, alphabetic	No tones, alphabetic
		Atypical language organization	Unlikely	Likely	Unlikely

## Data Availability

The author carefully documented all methods, materials, and data that were used to conduct the research presented in this article. The author agrees to share anonymized data upon request from any qualified investigator.
